# Insights into nitrogen allocation and recycling from nitrogen elemental analysis and ^15^N isotope labelling in 14 genotypes of willow

**DOI:** 10.1093/treephys/tpt081

**Published:** 2013-11-01

**Authors:** Nicholas J.B. Brereton, Frederic E. Pitre, Ian Shield, Steven J. Hanley, Michael J. Ray, Richard J. Murphy, Angela Karp

**Affiliations:** 1 Division of Biology, Imperial College London, South Kensington Campus, London SW7 2AZ, UK; 2 Institut de recherche en biologie vegetale, University of Montreal, 4101 Sherbrooke East, Montreal QC H1X 2B2, Canada; 3 Department of AgroEcology Rothamsted Research, Harpenden, Herts AL5 2JQ, UK; 4 Faculty of Engineering & Physical Sciences, Centre for Environmental Strategy, University of Surrey, Guildford, Surrey GU2 7XH, UK

**Keywords:** biofuel, biomass, nitrogen-use-efficiency, short rotation coppice willow

## Abstract

Minimizing nitrogen (N) fertilization inputs during cultivation is essential for sustainable production of bioenergy and biofuels. The biomass crop willow (*Salix* spp.) is considered to have low N fertilizer requirements due to efficient recycling of nutrients during the perennial cycle. To investigate how successfully different willow genotypes assimilate and allocate N during growth, and remobilize and consequently recycle N before the onset of winter dormancy, N allocation and N remobilization (to and between different organs) were examined in 14 genotypes of a genetic family using elemental analysis and ^15^N as a label. Cuttings were established in pots in April and sampled in June, August and at onset of senescence in October. Biomass yield of the trees correlated well with yields recorded in the field. Genotype-specific variation was observed for all traits measured and general trends spanning these sampling points were identified when trees were grouped by biomass yield. Nitrogen reserves in the cutting fuelled the entirety of the canopy establishment, yet earlier cessation of this dependency was linked to higher biomass yields. The stem was found to be the major N reserve by autumn, which constitutes a major source of N loss at harvest, typically every 2–3 years. These data contribute to understanding N remobilization in short rotation coppice willow and to the identification of traits that could potentially be selected for in breeding programmes to further improve the sustainability of biomass production.

## Introduction

An important target for achieving sustainable production of bioenergy is a reduction in agricultural inputs during the cultivation phase of the production process. In addition to providing increased energy security, the motive for producing bioenergy and biofuels from crops is to reduce the greenhouse gas (GHG) emissions that would otherwise be associated with fossil fuels. However, the use of nitrogen (N) fertilizer during cultivation requires energy (during manufacture) and can contribute to GHG emissions through nitrous oxide emissions (De Klein et al. 2006, Forster et al. 2007). Nitrogen fertilizer inputs for first-generation biofuel crops can be very high: 155 kg N ha^−1^ year^−1^ on average for US corn (ERS/USDA 2010) and 80–100 kg N ha^−1^ year^−1^ for Brazilian sugar cane (Martinelli and Filoso 2008); and 188 kg N ha^−1^ year^−1^ for UK wheat and 185 kg N ha^−1^ year^−1^ for UK oilseed rape (DEFRA 2010). In contrast, second-generation perennial biomass crops, such as rhizomatous grasses and fast-growing trees, have generally much lower N fertilizer requirements, resulting in more favourable energy and GHG balances (Karp and Shield 2008).

Willows (*Salix* spp.), grown as short rotation coppice (SRC), are among the important commercially grown biomass trees in temperate regions. Typical fertilizer inputs for SRC willow are low: 30–80 kg N ha^−1^ applied after cutback (following an initial establishment year) and after every harvest (every 2–3 years), giving average inputs of only 10–30 kg N ha^−1^ year^−1^ (Sennerby-Forsse 1995, Riche et al. 2010). Life cycle analysis performed by Miller (2010), which ranked the environmental impact of bioenergy feedstocks on the basis of land-use *and* N intensity (per 1000 GJ energy; such that the top ranking had the least environmental impact), placed SRC willow second after sugarcane, ahead of *Miscanthus*, sugar beet, oil palm, birch, poplar, switchgrass, corn, sweet sorghum and algae. Trials in willow that have compared different fertilization regimes (Bowman and Conant 1994, Labrecque et al. 1998, Weih and Nordh 2002) have indicated that a large spectrum of genotype-specific responses to fertilization exist, suggesting that there may be scope to improve this advantageous trait even further.

Short rotation coppice willow is known to recycle resources before the winter from leaves to stems, stools and roots to provide reserves for re-growth in the spring (Bollmark et al. 1999, Cooke and Weih 2005, Karp and Shield 2008). Seasonal N cycling is linked to phenology and is well characterized in *Populus* (Cooke and Weih 2005), a closely related genus to willow. Nitrogen is stored principally in vegetative storage proteins, particularly below the bark (bark storage proteins; BSPs). These show a characteristic pattern of accumulation in autumn and disappearance in spring within the perennating tissues of the bark, wood and roots. Rubisco in leaves, aside from its primary role in carbon fixation, is also thought to play a secondary role as a form of N storage (Cooke and Weih 2005). Three developmental stages are highlighted as of substantial importance during N cycling: (i) the initial growth phase and canopy establishment (June); (ii) the peak of the growth phase (August); and (iii) growth cessation and remobilization of resources before winter (October) (Bollmark et al. 1999, von Fircks et al. 2001).

Nitrogen assimilation, utilization efficiencies and N losses in SRC willow were previously studied by Weih and Nordh (2002) where, in the 14 genotypes tested, higher N utilization (there termed N productivity) had a strong positive influence on shoot biomass yield under N limiting conditions. There was also a general association between increased N assimilation and increased N losses. The major N losses from SRC willow are through leaf abscission in winter (Bollmark et al. 1999) and removal of the stems during harvesting (Sennerby-Forsse 1995). The N supplied through the leaf litter (Aerts 1996) and aerial N deposition (Fluckiger and Braun 1998) appears to suffice during inter-harvest years, but when the stems are harvested, a compensatory 30–80 kg of N fertilizer is required (Sennerby-Forsse 1995). There is also some evidence of low/no variation in rates of N uptake within the genus *Salix* (Ericsson 1981), although many studies do not clearly separate N uptake from utilization when addressing nitrogen-use efficiency (NUE). The limited understanding of the regulatory mechanisms for controlling N economy in willows and other crops are highlighted in two recent reviews addressing NUE (Hirel et al. 2007, Weih et al. 2011) from which it is clear that further insights are needed.

The main aim of the current study was to investigate how successfully different SRC willow genotypes assimilate and allocate N during establishment and the growing season as well as remobilize and consequently recycle N during leaf senescence before the onset of winter dormancy. To address this, allocation and remobilization of N to and between different organs were assessed in the parents and 12 progeny of a genetic mapping population using elemental analysis and ^15^N as a label. Our starting hypotheses were that: (i) genotypic variation occurs in primary N allocation with regard to canopy or root system establishment; (ii) shifts occur in the patterns of N allocation during the growing season in relation to biomass accumulation; and (iii) genotypic differences occur in allocation and remobilization of N between organs.

## Materials and methods

### Genotype selection

**Figure 1.**
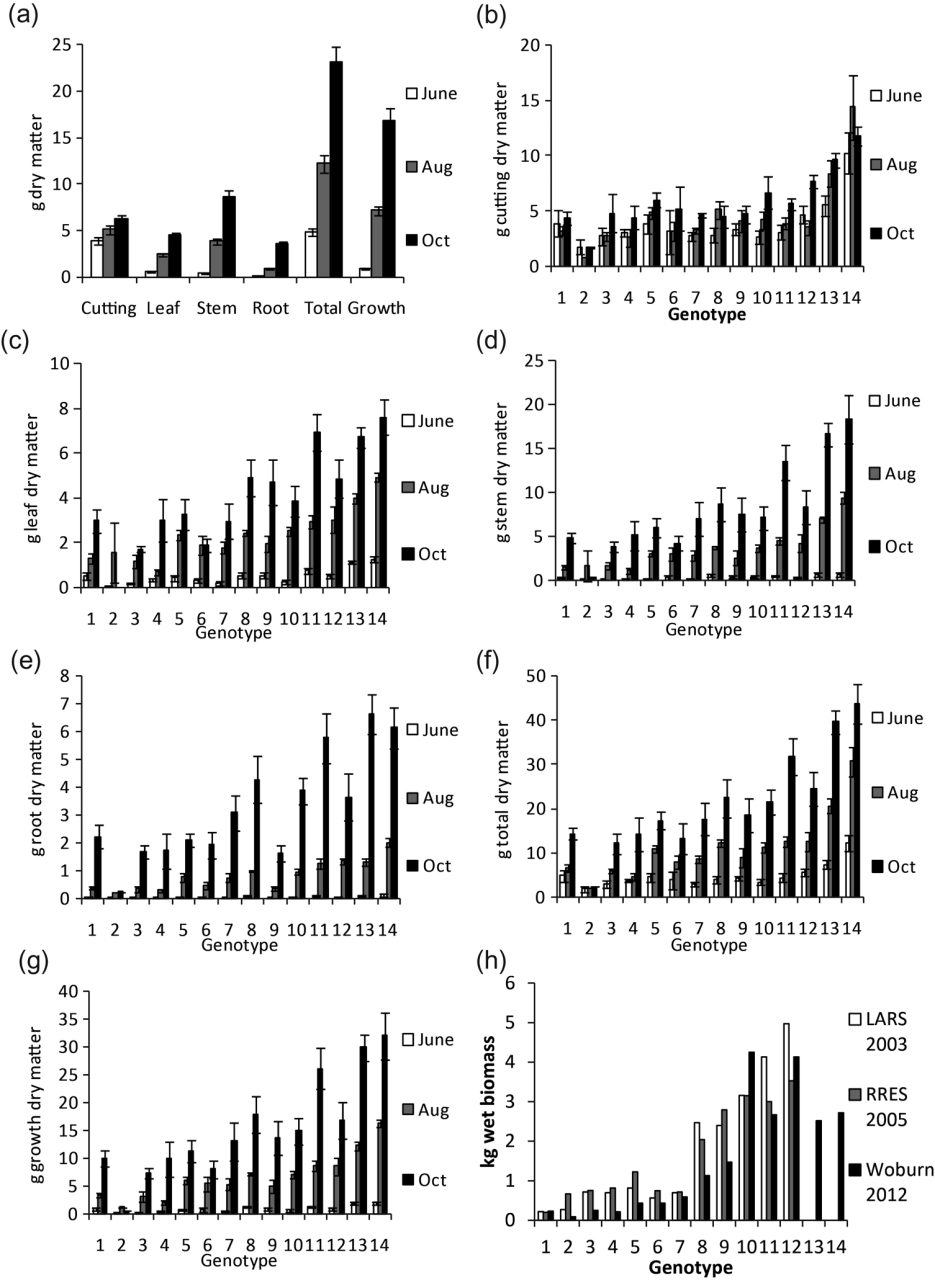
(a) Mean biomass yields (g DM) of all the trees at each harvest for each organ, total and growth (all organs with the exception of the cutting), error bars represent standard error (*n* > 60). (b–g) Mean biomass yield (g DM) for each genotype for each organ, total and growth (all organs with the exception of the cutting), for each genotype. The first, second and third columns represent values for the June, August and October harvests, respectively. Error bars represent standard error (*n* = 3–6). (h) Mean field, total above-ground, biomass yield (wet weight g), for each genotype the first, second and third columns represent values for LARS 2003, RRes 2005 and Woburn 2012 harvests, respectively. Genotypes 13 and 14 were not present in these field trials.

Cuttings were taken from the parents, ‘S3’ and ‘R13’ (here referred to as genotypes 13 and 14), and 12 progeny (referred to as genotypes 1–12) of a mapping population (K8) in which the parents S3 and R13 are two full-sib diploid *Salix viminalis* × (*S. viminalis* × *Salix schwerinii*) hybrids of grandparents, *S. viminalis* ‘Astrid’ and (*S. viminalis* × (*S. viminalis* × *S. schwerinii*)) (Hanley et al. 2006) (Table 1). The 12 progeny genotypes were specifically chosen because of the consistency and variation in their biomass yield as assessed over successive years at three field sites: Rothamsted Research (RRes, southeast England; 51°48′30′′N, 0°21′22′′W; 125m AOD), Long Ashton Research Station (LARS, southwest England; 51°25′22′′N, 2°40′12′′W; 50m AOD) and Woburn Experimental Station (Woburn, east England; 52°0′43′′N, 0°35′36′′W; 95m AOD). The recorded biomass yields from RRes (51°48′30′′N, 0°21′22′′W; 125m AOD) in 2005, LARS in 2003 and Woburn in 2012 were used to classify the 12 K8 genotypes into two separate yield groups, here termed ‘low’ (1–6) and ‘medium’ (7–12) yield group (Table 1, Figure 1h). The two parents of the population (13, 14) were also included, but yield data were only available from the Woburn site. Although the single-site field data for the two parents were similar to the genotypes categorized as medium yielding, the parents outperformed their progeny in the subsequent pot trials. The K8 population shows segregation for many important traits and has been aligned to the poplar genome sequence (Hanley et al. 2006) and used for mapping several QTLs in willow including saccharification potential (Brereton et al. 2010) and rust resistance (Hanley et al. 2011).

**Table 1.** SRC willow genotypes.

Pot trial genotype	Biomass yield group	Biomass yield LARS 2003 (kg wet)	Biomass yield RRes 2005 (kg wet)	Biomass yield Woburn 2012 (kg wet)
1	Low	0.22	0.20	0.23
2	Low	0.27	0.67	0.09
3	Low	0.72	0.75	0.25
4	Low	0.70	0.82	0.21
5	Low	0.82	1.22	0.43
6	Low	0.69	0.69	0.43
7	Low	0.57	0.57	0.59
8	Medium	2.46	2.04	1.13
9	Medium	2.40	2.79	1.47
10	Medium	3.16	3.15	4.25
11	Medium	4.13	3.00	2.67
12	Medium	4.97	3.53	4.13
13	Parent	–	–	2.52
14	Parent	–	–	2.72

The 12 genotypes from the K8 mapping population grown at RRes, LARS and Woburn Experimental Stations. Genotypes 13 and 14 are the K8 parents S3 and R13 and are two full-sib diploid *Salix viminalis × *(*S. viminalis × S schwerinii*) hybrids of grandparents, *S. viminalis* ‘Astrid’ and (*S. viminalis* × (*S. viminalis* × *S. schwerinii*)). Field wet annual biomass yields (kg). –, data not available.

### Growth conditions and experimental design

In April 2008, the cuttings were planted in 12-l pots filled with a 1 : 1 ratio of sand and perlite and placed outside in a roofed and caged area at RRes. Before planting, the 20-cm cuttings were first soaked in water overnight. There were 28 treatments (14 genotypes × 2 N treatments: with or without ^15^N) and three harvest dates replicated three times, giving a total of 252 individual pots of willow used in the experiment. These were arranged in a trial design comprising randomized blocks. The three harvests were taken in June, August and October after 2, 4 and 6 months of growth, respectively. Average hours of sunshine and max/min air temperature over the 2 months preceding each harvest were: 194 h 16.4/7.6 °C, 158 h 20.5/12.7 °C and 134 h 15.4/7.6 °C, respectively. The trees were fertilized with ammonium nitrate, either ^14^NH_4_^14^NO_3_ or ^15^NH_4_^15^NO_3_ (containing 10% ^15^N and 90% ^14^N). The natural abundance of ^15^N is 0.3676% and the fate of the 10% ^15^N applied was followed using isotope ratio mass spectrometry. Of the 252 potted cuttings, 45 failed to grow, of which genotypes 3 (8719) and 7 (8861) had the lowest number of survivors.

Fertilizer was applied in increasing amounts to correspond to the tree's ability to absorb and utilize N. By the first harvest 6 mg of N had been applied to each tree, 206 mg by the second harvest and 2.046 g by the final harvest. Nitrogen added between August and October was not enriched in ^15^N so that the remobilization of (the previously labelled) N during early senescence could be detected. Although lower total N applications per tree have been shown to be sufficient for the initial growth season of some willow varieties (von Fircks et al. 2001, Weih 2001, Weih and Nordh 2002), under field conditions the K8 population had grown to an average of 200 g dry matter (DM) during the establishment year (Hanley 2003). Using these data, and under the assumption that ∼1% of total DM biomass would be N (Debell and Radwan 1979, Chauvet 1987), an application regime of 2.046 g N in total was estimated as necessary N supply without exceeding requirements of the natural growth of the tree in the pots.

Each fertilizer application was made in 50 ml of water that also included a constant nutrient mix: (3 mM MgSO_4_ċ7H_2_O, 2 mM KH_2_PO_4_, 0.1 mM NaCl, 2 mM CaCl_2_ċ6H_2_O, 50 μM FeNaEDTA, 0.04 μM CuCl_2_ċ2H_2_O, 5.8 μM H_3_BO_3_, 1.145 μM MnCl_2_ċ4H_2_O, 0.065 μM Na_2_MoO_4_ċ2H_2_O and 0.1 μM ZnCl_2_ċ7H_2_O). The trees were given 200 ml of water every 2 days for the first 2 months. This was increased to 400 ml for the remaining 4 months.

### Sampled plant material

Each organ was destructively harvested; cutting, leaf, stem (i.e., new stem as opposed to the cutting), root, total and growth of biomass yields (g DM) were recorded for each individual after 2, 4 and 6 months (June/August/October). As the cutting represented a significant proportion of each tree's total weight, especially at the first harvest, the total growth in DM excluding the cutting is also presented here as ‘growth’ DM.

### Processing and stable isotope ratio mass spectrometry

At each of the three harvest time points, each of the four organs (cutting, leaves, stems and roots) from each tree was ground independently using a water cooled IKA^®^ A10 Analytical Grinder (IKA^®^ Works Inc., Wilmington, NC, USA). Prior to grinding, all samples were air-dried at room temperature for 2 days. A sample of 150 mg of ground biomass was then oven-dried over-night at 105 °C in order to assess the moisture content allowing the calculation of organ and total DM per tree. Then 100 mg of oven-dried sample from each organ was sent to Iso-Analytical Ltd Crewe, UK, for ^15^N stable isotope ratio mass spectrometry as well as total N assessment.

## Results

### Growth data and biomass yields

The first sign of growth from the cuttings was the appearance of new leaves. By June (after 2 months of growth), when all the trees were considered together as one group, 56% of growth DM (excluding the cutting) was leaf, only 38% was stem and 6% root. By August, the proportions had changed, with only 34% of the growth DM as leaf, 54% stem and 12% root. By the final harvest, in October, the tree biomass was 27% leaf, 52% stem and 21% root (Figure 1a). When analysed on an individual genotype basis, significant genotype differences (*P* < 0.05, ANOVA *F*-test) were revealed for the DM of each organ, as well as for total and growth DM at each harvest point (Figure 1b–g).

As the experiments were conducted in pots, the biomass yields were compared with known yield data for the same genotypes grown in the field. Mean genotype leaf, stem, root, total and growth biomass yields (g DM) from the pot trial, at both August and October harvests, had strong and significant positive correlations with the recorded field above-ground biomass yields from three field sites: RRes (harvested in 2005), LARS (harvested in 2003) and Woburn (Table 2, Figure 1h).

**Table 2.** Pearson**'**s correlations for field biomass yield and pot trial biomass yield traits.

	Cuttings (g)	Leaves (g)	Stem (g)	Roots (g)	Total (g)	Growth (g)
Harvest 2 August						
2003 LARS (kg)	0.439^a^	0.827	0.826	0.842	0.769	0.824
2005 RRes (kg)	0.484^a^	0.799	0.775	0.740	0.738	0.763
2012 Woburn (kg)	0.370^†^	0.768	0.755	0.782	0.695	0.757
Harvest 3 October						
2003 LARS (kg)	0.686	0.797	0.764	0.739	0.817	0.785
2005 RRes (kg)	0.646	0.755	0.698	0.629	0.748	0.714
2012 Woburn (kg)	0.740	0.621	0.595	0.654	0.693	0.630

All field weights are presented as total above-ground wet weight (kg) and all pot trial weights are presented as oven dry weight (g).

All correlations are significant *P* ≤ 0.05 (except for ^a^*P* ≤ 0.15 and ^†^not significant).

### Nitrogen uptake efficiency (UpE)

As little root growth (average 6% growth DM) had occurred by June (Figure 1e), the majority of N in the trees was presumed to be derived from the cutting, as opposed to being assimilated from the growth media (applied N). This was further verified using the stable isotope ^15^N ratio, which enabled quantification of assimilated N. The ^15^N ratio revealed <2% of total tree N had been assimilated from the growth medium (containing the labelled fertilizer) by June for all the trees. This cutting N (non-assimilated) was used as a genotype-specific baseline for tree UpE, which was subtracted from later harvests to establish assimilated N levels.

**Figure 2.**
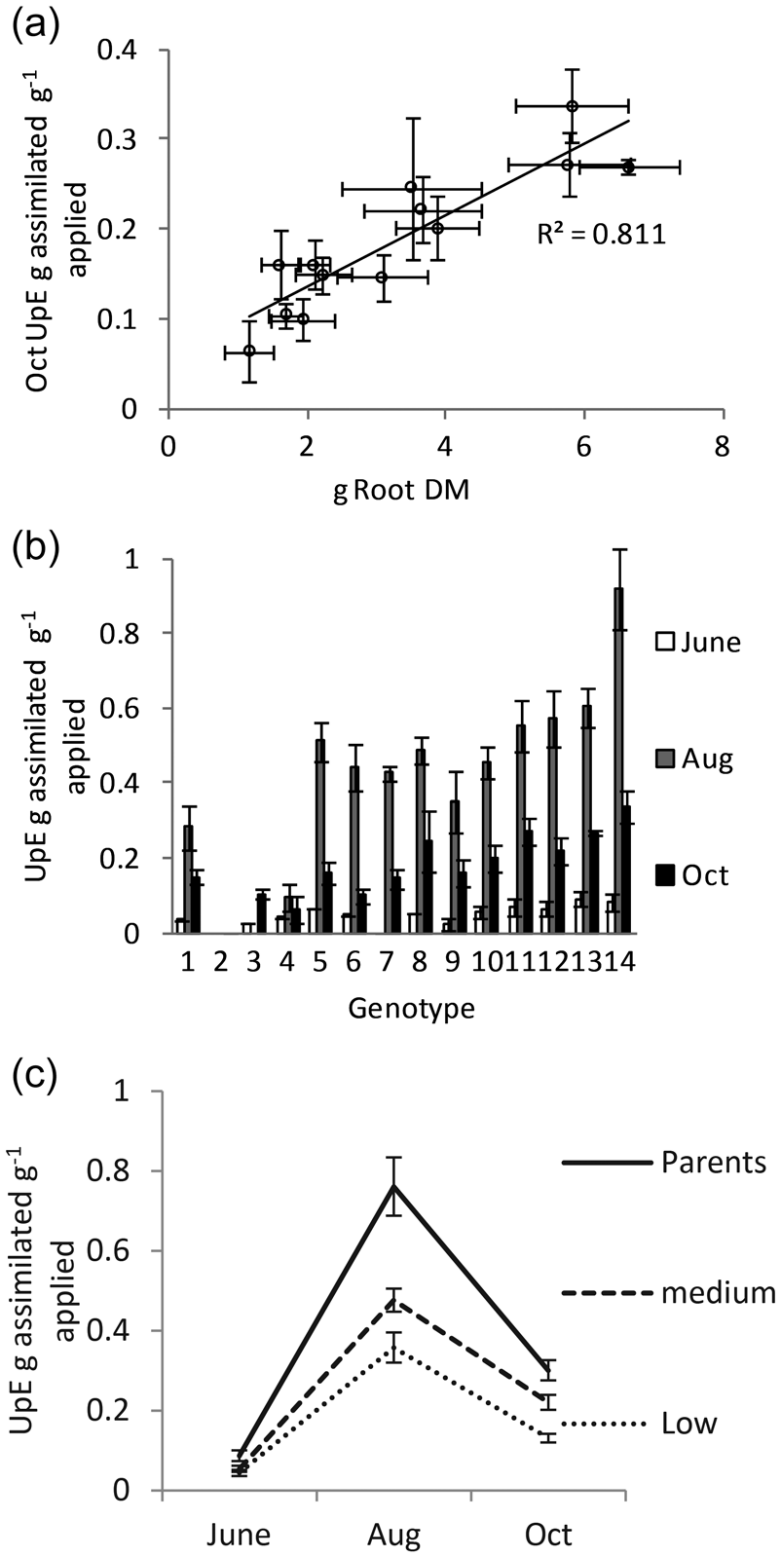
(a) Genotypic means of October root biomass yield correlated against UpE. Error bars represent standard error (*n* = 3–6). (b) UpE for each genotype. The first, second and third columns represent values for the June, August and October harvests, respectively. Error bars represent standard error (*n* = 3–6). (c) Mean biomass yield group UpE at June, August and October harvests. Error bars represent standard error (*n* = 12–30).

The UpE was found to strongly correlate to root DM at both the August and October harvests (October harvest—Figure 2a). A strong genotype effect was observed (Figures 1e and 2a and b). Genotype 14 had the highest root DM in August, second highest by October and the highest UpE in both August and October.

Once the genotypes were further categorized into biomass yield groups there was a clear and significant segregation for UpE (Figure 2c) (*P* < 0.05, ANOVA *F*-test). UpE was highest during August and the amount of available N was close to limiting for highest yielding genotypes, the parents, in August (between 70 and 80% assimilation of available N); however, this dropped sharply in October as fertilizer application was increased.

### Nitrogen primary allocation

**Figure 3.**
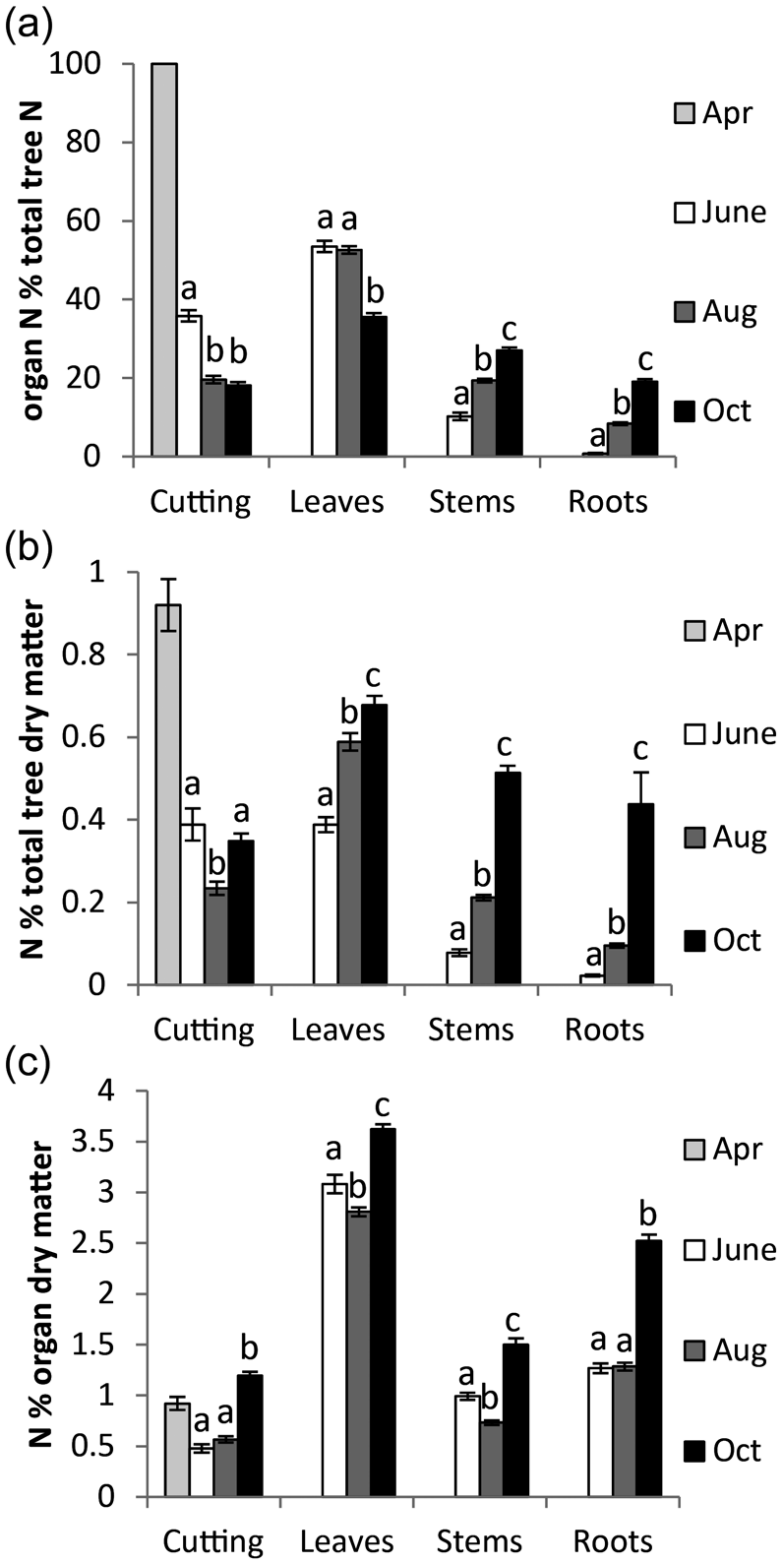
(a) Mean percentage of total tree N allocated to each organ. (b) Mean N in each organ as a percentage of the whole trees DM. (c) The N in each organ as a percentage of the organ DM. The first, second, third and fourth columns represent values for the April (indicating N allocation at the time of planting), June, August and October harvests, respectively. All error bars represent standard error (*n* > 60) and letters denote pairwise significant difference between harvests but *within* an organ (t-test, *P* < 0.05).

The proportion of the total tree N in each organ was calculated for every tree at each harvest point (Figure 3a). The initial mobilization of N from the cutting was largely to the leaves, which comprised ∼53% of total tree N by June, with only 10% to the stem. In the following two harvests the overall amount of N vastly increased both in absolute amounts (concurrent with increased whole tree growth) and proportionally in relation to DM (Figure 3b and c). The allocation pattern observed in June was not maintained through to August and October (Figure 3a) when total N allocated to both the stem and roots of the trees increased in August to 19 and 8%, respectively, and in October to 27 and 19%, respectively. The initial investment of total N to the leaves was maintained in August, even after the shift of N source from cutting reserves to recently assimilated N from the growth medium. However, by October the proportion of total N allocated to the leaves had dropped substantially from 53 to 35%.

**Figure 4.**
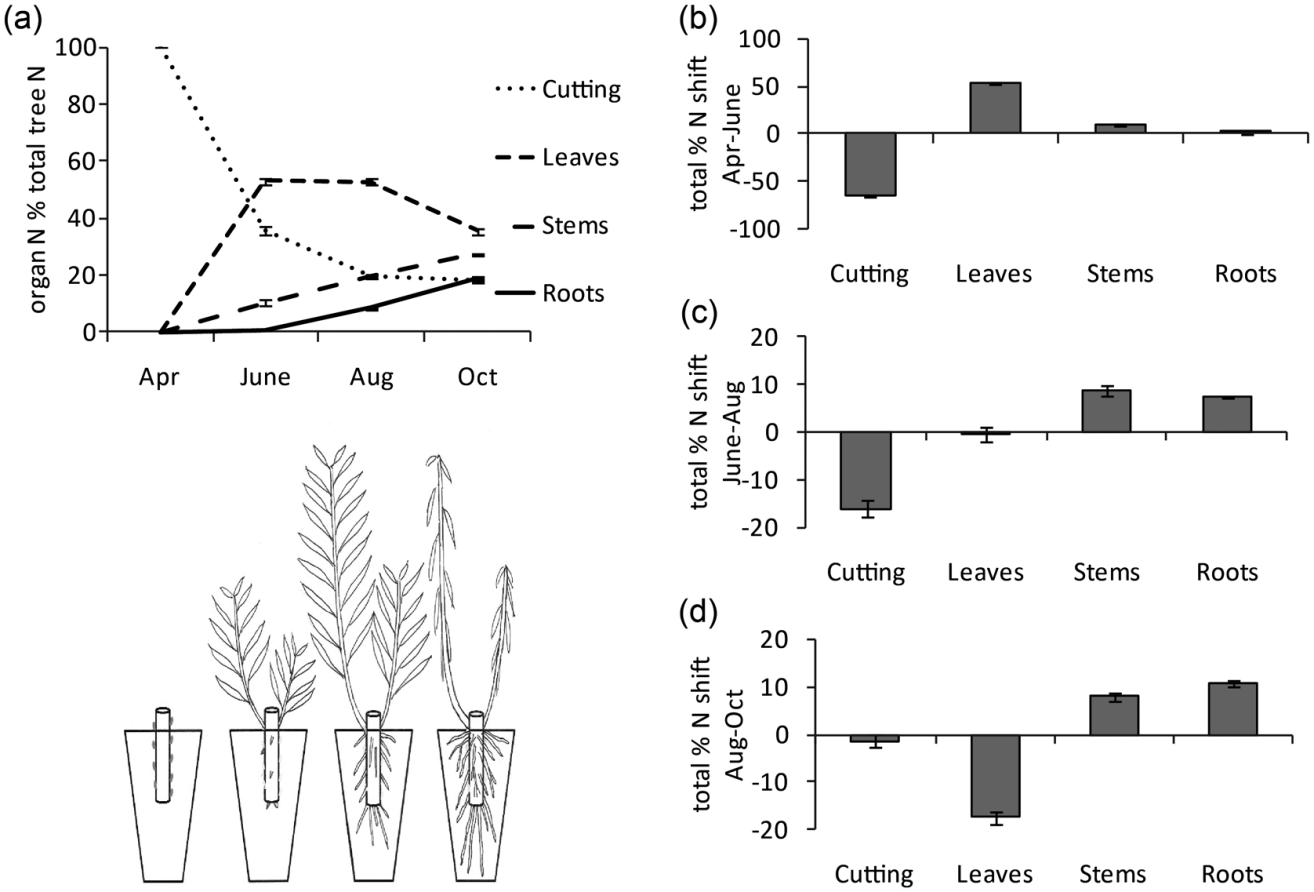
(a) Schematic of plant development over the period April to October. The far left pot shows the planted cutting. The graph above traces mean total N allocation to each organ over the same period. Error bars represent the standard error (*n* > 60). (b–d) Mean shift of total N allocation to each organ between April and June, June and August, and August and October. Error bars represent the standard error of the difference between the two means (*n* > 60).

To specifically interpret these changes in N ‘strategy’, with relation to the growth of the trees, the data are also presented as the shift between sampling points (Figure 4a–d). Between April and June > 64% of the N reserves in the cutting were remobilized, with the majority going to leaves, only a small amount going to the stem and little to no N allocated to the roots. The investment in stem and roots increased from June to August by 9 and 8% of the trees total N, respectively. This trend of increased importance of stem and root N continued with further proportional increases between August and October at the expense of allocation of assimilated N to leaves, resulting in a reduction of over 17% of total N to the leaves.

**Figure 5.**
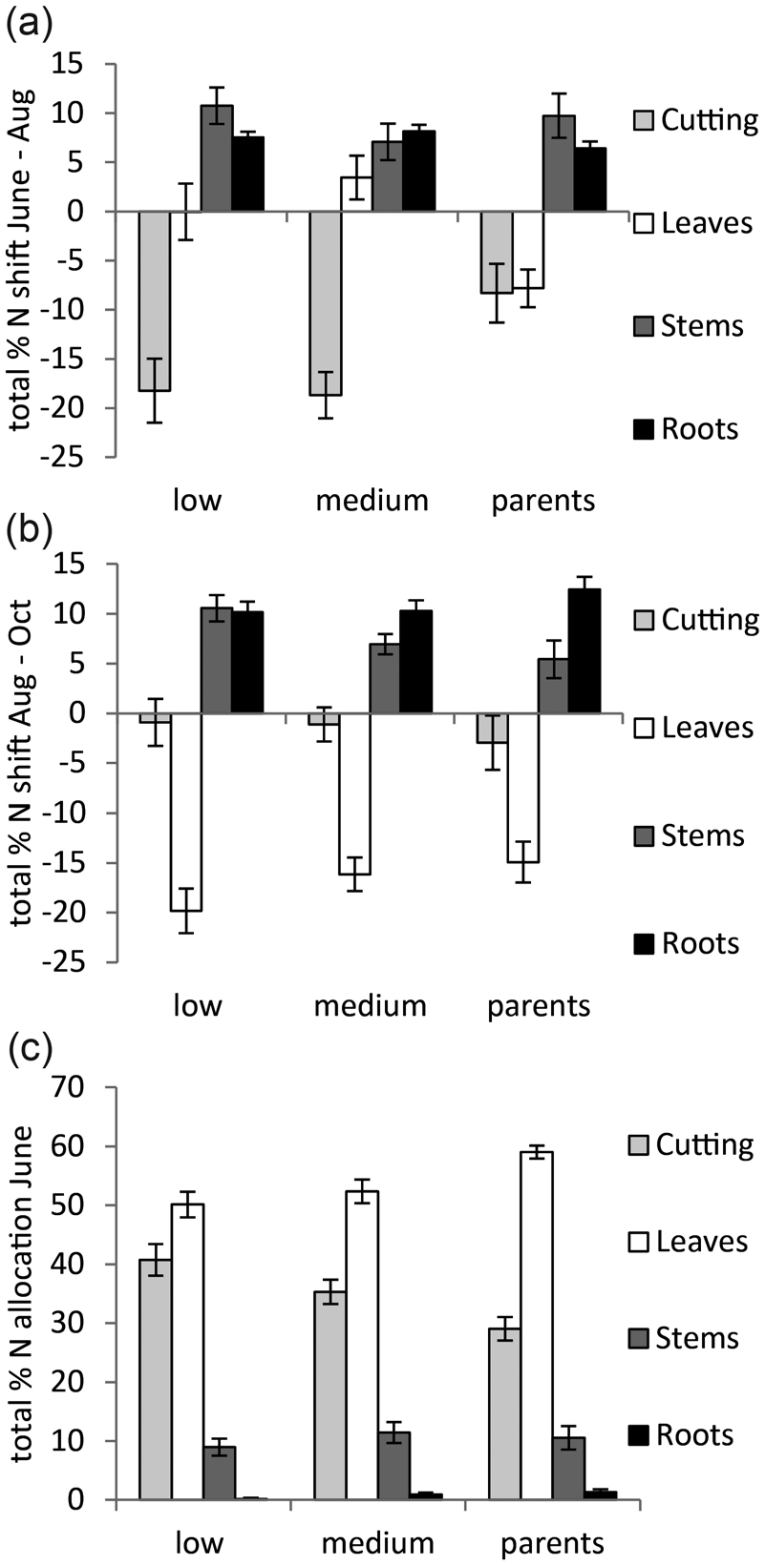
(a and b) Mean shift of total N allocation to each organ between June and August and August and October for each biomass yield group: low, medium and parents. Error bars represent the standard error of the difference between the two means (*n* = 12–30). (c) Mean total N allocation to each organ in June for each biomass yield group; low, medium and parents. Error bars represent the standard error (*n* = 12–30).

However, when the data were considered after categorizing the genotypes into the biomass yield groups, distinct differences in the shifts in allocation could be seen. The largest difference occurred between June and August for the parents, where there was a marked reduction in N mobilization away from the cutting and a reduction in allocation to the leaves (Figure 5a). These same shifts were seen later in the other two groups, during August and October (Figure 5b). The difference in allocation shown by the parents was reflected by their earlier utilization of the cutting resources (or its faster depletion), since by June only 29% of the total N was present in the cutting, in contrast to 35% for the medium yielding group and 40% for the low yielding group (Figure 5c).

### Nitrogen remobilization

Isotope labelling with ^15^N was used to quantify assimilated N and to follow N remobilization as distinct from primary allocation. As the enrichment of fertilizer with the ^15^N ceased after the August harvest, it was possible to follow remobilization of N between August and October.

**Figure 6.**
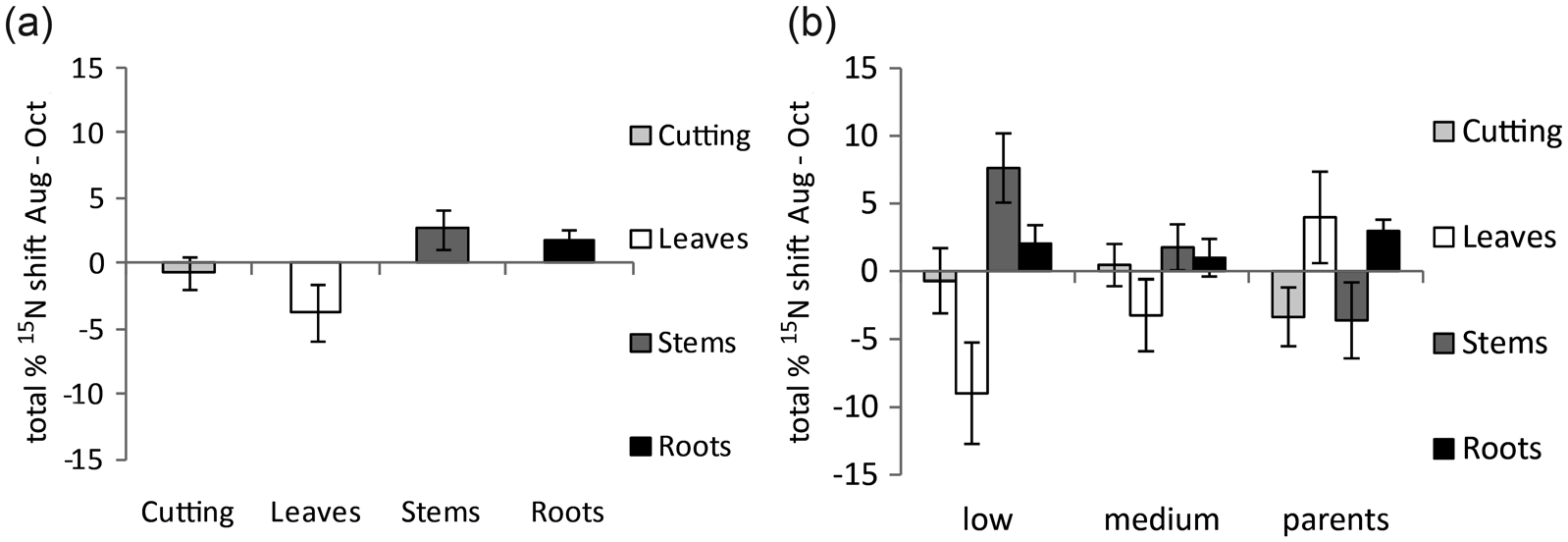
(a) Mean shift of total ^15^N to each organ between August and October. All trees from all genotypes are pooled. Error bars represent the standard error of the difference between the two means (*n* > 60). (b) The mean shift of total ^15^N between organs of all the trees between August and October. Trees are grouped into biomass yield groups. Error bars represent the standard error of the difference between the two means (*n* = 12–30).

Looking at the grand mean of all genotypes together, there was no substantial remobilization of N between August and October from the cutting and only small movements between the other organs, with 2.5 and 1.5% of total assimilated N mobilized to the stems and roots, respectively (Figure 6a). Remobilization of N towards both stem and root came at the expense of a 4% remobilization away from the leaves. When trees were categorized according to their biomass yield groups, significant variation was observed (*P* < 0.05, ANOVA *F*-test); low yielders had a larger N remobilization *away* from leaves (9%) and *into* stems (7.5%), whereas medium yielders had a reduced difference with only a small remobilization of N *away* from leaves (3%) and *into* stems (1.5%). The two parental genotypes, here the highest biomass yielding, showed the reverse, with a remobilization of N *into* the leaves (4%) and *away* from the stems (3.5%) (Figure 6b).

## Discussion

In the present study, ^15^N was used a label to investigate allocation and remobilization of N in different SRC willow genotypes from cutting establishment up to leaf senescence, before the onset of winter dormancy. It was hypothesized that genotypic variation in primary N allocation would occur in relation to canopy or root system establishment, that shifts in the patterns of N allocation relating to biomass accumulation would take place during the growing season, and that genotypic differences would be present in allocation and remobilization of N between organs. Genotype-specific variation was observed for all traits measured and all three hypotheses were supported by the results found.

### Pot growth data and validation

The pot trial was grown outside in a covered cage (and not in glasshouse conditions) using a defined supply of fertilizer that was intended to approximate field conditions as much as possible. In pot conditions, relative biomass yields by genotype were similar to those obtained in the field. Overall, correlations were comparable to (or even stronger than) those previously reported in willow (Table 2) (Lindegaard et al. 2001, Weih and Nordh 2005, Weih and Bonosi 2009). Moreover, the first harvest cycle of SRC willow often has reduced yields, yet significant correlations were maintained between the pot-grown trees and the corresponding genotypes grown in the field at the second harvest cycle (Figure 1h). This suggests that, for biomass yield at least, the pot system reflected the field after 7 years of root growth (including two, 3 year stem harvests).

As expected growth DM steadily increased over the three harvest points; however, the allocation of mass to each organ showed a more complex pattern. By the first harvest in June the majority of the trees' resources had been committed to producing leaves and there was very little or no observable root development. When considered in conjunction with the small amount of growth medium-derived (assimilated) N in June, there is strong evidence that the cutting acted as the major source of N for that entire period of growth. By June the majority of the trees' resources had been committed to producing leaves (Figure 1). Weih and Nordh (2005) found a similar relationship in a 6-month pot trial growing willow from cuttings, where increases in leaf area were also seen at the cost of resource allocation to the root biomass, further supporting the crucial role the cutting plays during this initial growth period.

Some work has been performed showing that both increased diametre and/or length of a planted cutting can have a positive impact on subsequent growth performance in a genotype-specific manner (Shield et al. 2008). However, there is scope for cutting optimization, especially when considering that seasonal mobilization of resources between above- and below-ground biomass may result in variation in stem nutrient content (and consequently impact the cuttings made from them).

By August, a shift from a high growth rate of leaves to a high growth rate of stems was seen as gradually the stems became the largest organ of the tree (Figure 1a). Prioritization of the stem had even further increased by October, when an increase in root development was also observed. These simple general trends in growth help confirm the distinct developmental points of interest chosen.

### Nitrogen UpE

It would seem evident that the initial low rate observed for UpE (Figure 2e, <0.1 g assimilated g^−1^ available) resulted from reduced root development in June, initially marginalized as a consequence of the principal investment into canopy establishment. This relationship is further supported by the strong correlation, observed between root development and UpE in both August and October (Figure 2a). A similar correlation was previously reported for pot-grown willow by Rytter (2001, Rytter and Hansson 1996). Our aim of applying increasing amounts of N without resulting in excess was attained by the August harvest point, but the sharp decline in UpE at the October harvest indicates that the applied N was then in excess. Only the two parents had close to 100% N assimilation by the August harvest and were therefore possibly limited by N available in the growth medium. Although it was clear that these two willow genotypes had greater uptake capacity here, the system is limited by providing only a single form of N for assimilation. As different forms of N can result in greatly different responses from trees (in terms of uptake) (Domenicano et al. 2011), it would be interesting to see whether the trend held for these different genotypes when other forms of N are available and to what degree, if any, this variation may contribute to genotypic variation in affinity for different field environments.

### General N allocation

A clear sequence of developmental steps was evident in the timing of N allocation (Figure 4a). There was an initial large commitment of the N reserves to leaves, indicating that priority was first given to canopy development and carbon fixation after which allocation to root development and soil nutrient assimilation followed.

There was a substantial reduction in the proportion of total tree N in the leaves by October and large increases of N investment into both stem and root, which suggest that a strategic shift in developmental priorities occurred at this time. It was not possible to determine to what extent the shift was due to remobilization or a change in primary allocation, based on the observed N content alone. This is because of the large increases in both biomass and N content that occurred within the tree between August and October; however, due to the ^15^N labelling strategy used, it was later possible to separate N remobilization and primary allocation.

### Nitrogen allocation (by genotype and biomass yield group)

The parental genotypes showed a distinct reduction in allocation of N from the cuttings between June and August, which could be due to earlier utilization or depletion of the cutting resources. Interestingly, this June–August shift from cutting reliance resembled the same shift observed between August and October in medium- and low-yield groups (Figure 5a and b). This could be indicative of an earlier or more successful canopy establishment, consequently leading to the trees investing earlier in roots and stems (Figure 5c).

Strong evidence between the relationship of earlier canopy establishment (through early bud burst) and increased biomass production has previously been demonstrated in willow by Rönnberg-Waestljung and Gullberg (1999) and Weih (2009). The alternative explanation is that depletion of the N resources in the cutting led to the change from prioritizing canopy establishment towards root development (and thus increased soil N assimilation).

Seasonal storage and mobilization is well characterized in SRC willow for starch, the plant's primary non-permanent carbon store, with the roots being the major storage organ over winter dormancy (Von Fircks and Sennerby-Forsse 1998). As evidence supports that the major N storage organ over winter is the stem (von Fircks et al. 2001), it is not surprising that the cutting, which effectively changes from being an above-ground to a below-ground organ once it is planted, is high in N (instead of starch) and that canopy development and carbon fixation is an even greater priority in the new growth from the planted cutting than occurs in normal re-growth in spring. This resource balance, as a result of vegetative propagation, would also support the late shift (between August and October) from initial carbon limitation to N limitation and consequent large increase in prioritization of root development.

### Nitrogen remobilization

A small but clear trend became apparent when data from all the trees were pooled (Figure 6a), of N moving from leaves to the stem and a lesser amount to the roots. However, separation of the trees into their biomass yield groups showed that reduced remobilization of N from the leaves to the stem (between August and October) was linked to increased biomass yield (Figure 6b). There is a much more stark difference than that seen when observing primary allocation alone and could be the result of internal resource regulation that significantly influences the length of the growing season. However, although the difference in remobilization is significant at this point in time (October), it is not possible to discount variation beyond this point, which would require additional harvest points after the completion of leaf senescence, and thus the data should be viewed as preliminary. This finding is in contradiction with that of a pot trial conducted by Weih (2001), where a higher biomass yielding genotype (‘Tora’) had greater N remobilization away from leaves during autumn than a lower biomass yielding genotype (‘L78183’), but in agreement with many recent findings demonstrating that longer leaf duration can result in a large increase in biomass production (Weih 2009).

Another interesting finding revealed by the ^15^N data is that remobilization of resources seemed to be primarily a shift from leaves to stems, with only a small amount being remobilized from above-ground biomass to below-ground biomass (Figure 6a and b). Previous studies have also provided evidence for preference to above-ground N storage (Bollmark et al. 1999). This raises an interesting possible dilemma concerning sustainability. Whilst high N reserves in the stem seem important for the establishment of new plantations from propagated cuttings, they would conversely be considered a disadvantage if the stems are destined for thermal combustion. Moreover, if agricultural inputs are to be kept at a minimum (De Klein et al. 2006, Forster et al. 2007), a reduction in stem N would also lead to less off-take of N at harvest and thus lower requirements for fertilizer application in the following spring. Consequently, the efficiency of *N retention*, the trees ability to reduce N loss through outputs such as leaf abscission, could potentially include the trees ability to mobilize N from above-ground (harvested) to below-ground (non-harvested) biomass over a pre-harvest winter if subsequent harvest yields are to be maintained while minimizing agricultural land inputs. The potential physiological benefits for substantial N remobilization from stems to roots, which is well understood in grasses such as *Miscanthus* where the rhizome is the perennial organ (Finch et al. 2009), are less apparent in SRC willow, where any reduction in winter stem N could potentially have deleterious effects on early re-growth in spring.

It has previously been speculated that N reserve formation is supported in two ways: a reduction in the growth-related N sink and in remobilization of N away from senescing leaves (Bollmark et al. 1999). The two factors observed here, (i) a high amount of N remobilization to below-ground biomass is important for reducing agricultural inputs and (ii) a low amount of N remobilization away from leaves was associated with high biomass yields, could lead to an interesting point of divergence for genotype selection.

The form of N was not directly assessed here and, since clear differences in the timing of remobilized N were identified, it is possible that genetic variability in the form of N-containing compounds may also be present. Further work addressing such variation should focus on winter storage compounds such as BSPs as well as proteases and amino acid transporters. Research has identified a number of BSP genes where transcript abundance correlates with natural total N content in poplar bark and also differentially responds to varying N application (Cooke et al. 2003, Cooke and Weih 2005, Wildhagen et al. 2010). In addition, a genetic basis to seasonal BSP accumulation has been reported among different clones of poplar. Significant differences in BSP accumulation occurred among four out of the six poplar full-sib families examined (*Populus trichocarpa* Torr. and Gray × *Populus deltoides*: three F_2_ families, two F_1_ families and one BC_1_ family). Bark protein and bark N concentrations, which also varied significantly between clones within families, were positively correlated to BSP amounts within several of the families (Black et al. 2001). It would be interesting to establish whether the genotypic variation identified in the present study has a similar basis as the results could have significance in selecting for clones with improved N storage capacity and NUE.

## Conclusions

The combined use of elemental analysis and ^15^N as a label enabled insights to be gained on N allocation and N remobilization (to and between different organs) in 14 genotypes of a genetic family. Initial canopy establishment was almost entirely fuelled by resources remobilized from the cutting, showing that the cutting acted as a primary resource hub for ∼8 weeks after planting. Root biomass accumulation and N assimilation (UpE) were strongly associated and varied between genotypes but an *earlier* cessation of the dependency on the cutting for N was linked to higher biomass yields.

Through observation of both organ biomass accumulation and N allocation, a clear sequence of growth priorities was identified. In the first 2 months, canopy establishment took precedence over root development yet after this time, long before leaf senescence, resource investment shifted towards the stems and roots. Earlier canopy development, in conjunction with reduced (or delayed) N remobilization from the leaves to the stems and roots by October, resulted in increased biomass yields. This provides supporting evidence that increased canopy *duration* over the season is a major factor in biomass accumulation.

Little variation in root N allocation was observed although root biomass production was highly varied. Evidence was also found that the stem is the major N reserve during winter dormancy of SRC willow. To increase *N retention*, with the hope of reducing agricultural land inputs and opening up more low-nutrient land for cultivation, research should be directed towards further increasing the roots or stools as N sinks before harvest without deleteriously effecting inter-harvest bud burst in spring.

## Conflict of interest

None declared.

## Funding

The authors gratefully acknowledge the funding support from the Rothamsted Cropping Carbon Institute Strategic Programme Grant and from the Porter Alliance (http://www.porteralliance.org.uk/) for a studentship awarded to N.J.B.B. F.E.P. was supported through a FQRNT postdoctoral research fellowship from the Government of Quebec, Canada.
